# Metagenomic sequences from anaerobic chloroform and dichloromethane degrading microbial communities

**DOI:** 10.1128/mra.00391-24

**Published:** 2024-07-01

**Authors:** Olivia Bulka, Elizabeth A. Edwards

**Affiliations:** 1 Department of Chemical Engineering and Applied Chemistry, University of Toronto, Toronto, Ontario, Canada; Montana State University, Bozeman, Montana, USA

**Keywords:** anaerobic dechlorination, chloroform, dichloromethane, metagenomes

## Abstract

Here, we present metagenomes from two cultures derived from an anaerobic microbial consortium used for bioremediation. One culture dechlorinates chloroform to dichloromethane, which is further mineralized to CO_2_. A second subculture was amended with only dichloromethane. We sought draft genomes of key microorganisms to identify metabolic potential in these consortia.

## ANNOUNCEMENT

SC05 is an enrichment culture used for bioremediation (also known as KB-1 Plus CF) originally derived from contaminated groundwater and maintained at SiREM in Guelph, ON, Canada (https://www.siremlab.com/). It reductively dechlorinates chloroform (CF) to dichloromethane (DCM), which is further transformed into carbon dioxide and hydrogen (1). A subculture, named SC05-UT, has been maintained at the University of Toronto on CF alone [~1 mM (aqueous)/2–4 weeks] without an external electron donor since 2018, where the hydrogen produced from DCM serves as the electron donor for CF dechlorination (2). A second subculture, named DCME, was transferred from SC05-UT and has been enriched by feeding DCM alone since 2019 [~1 mM (aqueous)/2–4 weeks] (2). *Dehalobacter* was implicated in CF dechlorination and DCM mineralization, but the cultures also harbor a variety of other microorganisms, 20–30 of which exist at an abundance >1% based on 16S amplicon sequencing (2). These metagenomic data were collected to understand the metabolic mechanisms of tandem CF and DCM degradation in this complex microbial community.

**Fig 1:**
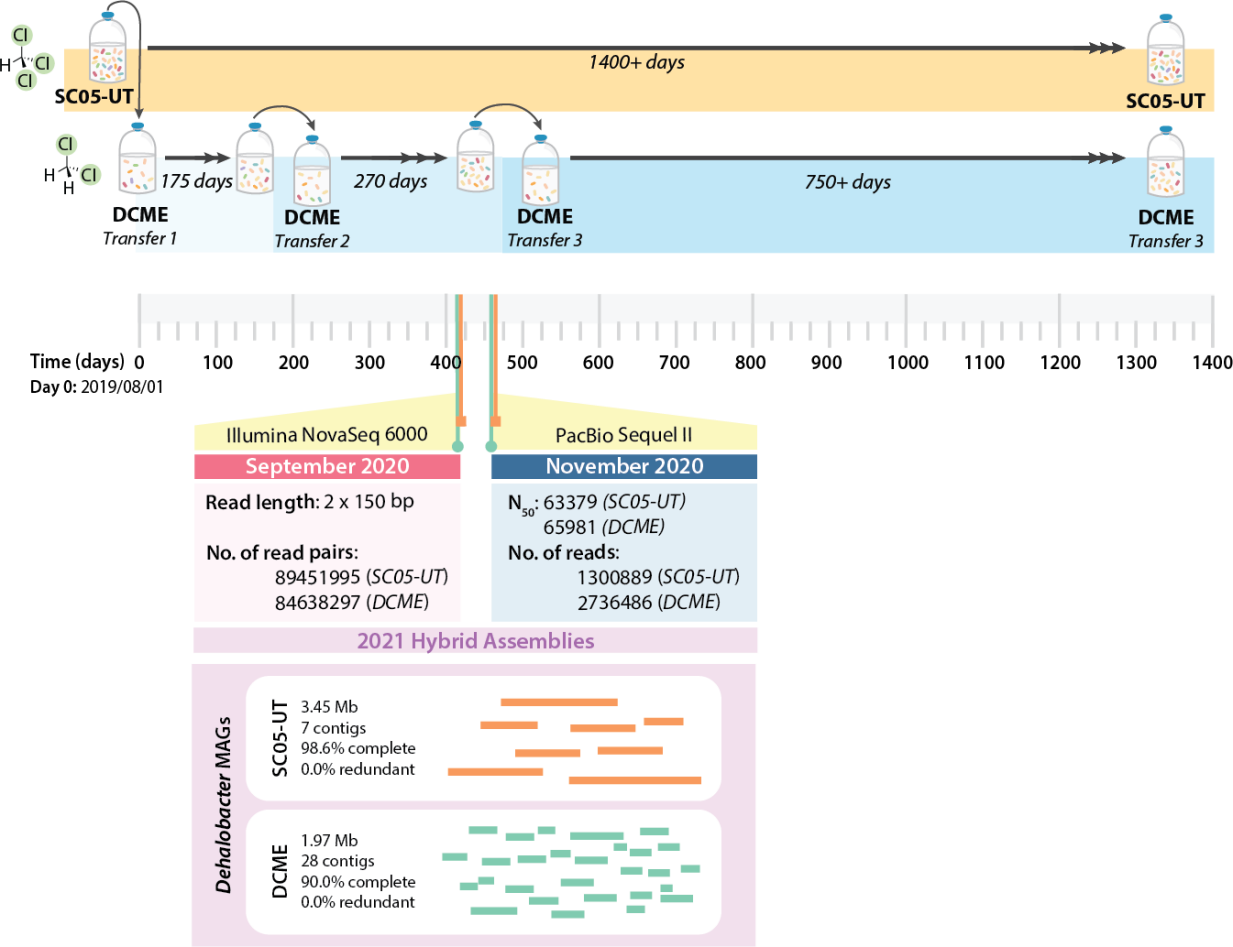
Summary of experimental setup and sequencing performed in this study. DNA samples are noted on the timeline (SC05-UT: orange squares, DCME: green circles) and labeled with the type of sequencing and sequencing stats. NovaSeq paired-end sequencing was performed in September 2020 (pink), while PacBio long-read sequencing was performed in November 2020 (blue). Co-assembly of both read types was performed for each culture, resulting in one *Dehalobacter* MAG each (purple).

These methods are expanded versions of descriptions in our related work (3). SC05-UT and DCME were sampled in September and November 2020 (SC05-UT: 300 mL, DCME: 400 mL) for a total of four samples (Fig. 1). DNA was extracted using the Kingfisher Duo Prime MagMax Microbiome Kit (Thermo Scientific, Waltham, MA, USA). Sequencing was performed by the Genome Quebec Innovation Centre (Montréal, QC, Canada). The library from each September sample was prepared using the NEBNext Ultra II DNA Library Prep Kit for Illumina (New England BioLabs, Ipswich, MA, USA), which includes PCR enrichment before Illumina NovaSeq 6000 sequencing (Table 1). Reads were quality controlled using FastQC v0.11.9, and the first three low-quality bases were trimmed using Trimmomatic v0.39 (4, 5). The November samples were prepared using the SMRTbell Express Template Prep Kit 2.0 (Pacific Biosciences, Menlo Park, CA, USA) without shearing or size selection for PacBio Sequel II sequencing (Table 1). Illumina and PacBio reads from each subculture were pooled for co-assembly with hybridSPAdes (SPAdes v3.15.0, python v3.6.12) (6) with all software using default parameters. Additional quality control, mapping, and binning were performed within Anvi’o v7 for contigs >1 kb (7–9), using MetaBAT2 v2.15, MaxBin v2.2.7, and CONCOCT v1.1.0, and ultimately DAS Tool v1.1.2 for dereplication and selection of bins >50% complete and <50% redundant (10–13). Taxonomy of each metagenome-assembled genome (MAG) was estimated using GTDB v89.0 (14, 15).

**TABLE 1:** Summary of sequencing reads and metagenomes reported in this study

Culture	Sequencing platform	Read length (bp)^*a*^	Number of reads	Insert size (bp)	Assembly size (Mb)	Number of contigs	Accession (SRA, Genbank)
SC05-UT	Illumina NovaSeq 6000	150	82,420,701 (×2)	250	413.5	467,998	SRX21660399, JAWDGN000000000
	PacBio Sequel II	63,379	1,300,889	NA			SRX21660401, JAWDGN000000000
DCME	Illumina NovaSeq 6000	150	77,791,279 (×2)	250	243.5	200,127	SRX21660400, JAWDGO000000000
	PacBio Sequel II	65,981	2,736,486	NA			SRX21660402, JAWDGO000000000

^*a*^ For PacBio, this refers to *N*_50_ of reads.

Forty draft MAGs were recovered from DCME, and 51 from SC05-UT, representing 53% and 77% of total microorganisms, respectively, as determined by relative abundance estimates in Anvi’o (mean completion = 85.5%, mean contamination = 5.9%) (7). One *Dehalobacter* MAG was assembled from SC05-UT (7 contigs, *N*_50_ = 571,539) and DCME (28 contigs, *N*_50_ = 148,192). Other MAGs included several Archaea (5/7, DCME/SC05-UT), and many bacteria, including Anaerolineales (5/9, DCME/SC05-UT), Synergistales (5/6, DCME/SC05-UT), and Bacteroidales (3/1, DCME/SC05-UT). Difficulties arising from uneven paired-read coverage from PCR enrichment prior to sequencing precluded any closed genomes from this assembly. We are working to refine the *Dehalobacter* MAGs with further sequencing attempts (O. Bulka and E. A. Edwards, unpublished data) and analyze the metabolic capacity of these cultures through annotation of MAGs (3).

## Data Availability

All reads and assemblies are available on NCBI under BioProject PRJNA1013980. This Whole Genome Shotgun project has been deposited in GenBank under the accession no. JAWDGN00000000 (SC05-UT) and JAWDGO00000000 (DCME). The version described herein is the first version, JAWDGN01000000 and JAWDGO01000000. Illumina paired reads have been deposited in the Sequence Read Archive under accession numbers SRX21660399 (SC05-UT) and SRX21660400 (DCME). PacBio reads were deposited in the SRA under accession numbers SRX21660401 (SC05-UT) and SRX21660402 (DCME). A summary of the quality statistics and taxonomy of each draft MAG is available on FigShare at 10.6084/m9.figshare.25460641. Fasta files of all draft MAGs in the DCME and SC05-UT metagenomes are available at 10.6084/m9.figshare.25057760 and 10.6084/m9.figshare.25057766, respectively.
